# Multitask training promotes automaticity of a fundamental laparoscopic skill without compromising the rate of skill learning

**DOI:** 10.1007/s00464-015-4713-9

**Published:** 2016-01-07

**Authors:** Jamie M. Poolton, Frank F. Zhu, Neha Malhotra, Gilberto K. K. Leung, Joe K. M. Fan, Rich S. W. Masters

**Affiliations:** 1 Carnegie Faculty, Leeds Beckett University, Leeds, UK; 2 Institute of Human Performance, University of Hong Kong, Pokfulam, Hong Kong; 3 Department of Surgery, Li Ka Shing Faculty of Medicine, University of Hong Kong, Pokfulam, Hong Kong; 4 Faculty of Education, University of Hong Kong, Pokfulam, Hong Kong; 5 Te Oranga School of Human Development and Movement Studies, University of Waikato, Hamilton, New Zealand

**Keywords:** Surgical education, Surgical skills training, Multitasking, Automaticity, Intraoperative stressors

## Abstract

**Background:**

A defining characteristic of expertise is automated performance of skills, which frees attentional capacity to better cope with some common intraoperative stressors. There is a paucity of research on how best to foster automated performance by surgical trainees. This study examined the use of a multitask training approach to promote automated, robust laparoscopic skills.

**Methods:**

Eighty-one medical students completed training of a fundamental laparoscopic task in either a traditional single-task training condition or a novel multitask training condition. Following training, participants’ laparoscopic performance was tested in a retention test, two stress transfer tests (distraction and time pressure) and a secondary task test, which was included to evaluate automaticity of performance. The laparoscopic task was also performed as part of a formal clinical examination (OSCE).

**Results:**

The training groups did not differ in the number of trials required to reach task proficiency (*p* = .72), retention of skill (*ps* > *.45*), or performance in the clinical examination (*p* = .14); however, the groups did differ with respect to the secondary task (*p* = .016). The movement efficiency (number of hand movements) of single-task trainees, but not multitask trainees, was negatively affected during the secondary task test. The two stress transfer tests had no discernable impact on the performance of either training group.

**Conclusion:**

Multitask training was not detrimental to the rate of learning of a fundamental laparoscopic skill and added value by providing resilience in the face of a secondary task load, indicative of skill automaticity. Further work is needed to determine the extent of the clinical utility afforded by multitask training.

A major threat to competence, particularly of trainees, is the diverse array of stressors that surgeons encounter in the operating environment [1]. This has motivated authorities in surgical education to seek to embed empirically tested training programs in the surgical curriculum [2]. The design of effective surgical training programs calls for an understanding of the psychomotor makeup of experienced surgeons [3], as proficient performance in the face of intraoperative stressors is a hallmark of surgical expertise.

Expertise approaches to skill learning aim to systematically identify factors that distinguish experts from their less skilled counterparts. Automaticity of performance is considered in non-surgical [4] and surgical domains [5] to be an attribute that defines expertise. Automaticity is referred to here as proficient performance of a skill with minimal support from conscious control processes [6] that typically are engaged during earlier stages of learning [7]. One index of the automaticity of technical skills is the capability of the performer to concurrently handle attention-grabbing secondary tasks without disruption of primary task performance [8]. Surgeons with extensive laparoscopic experience, for example, have been shown to be able to attend to a secondary visual detection and recall task while executing proficient intracorporeal sutures and knot ties; however, technically proficient trainees with limited laparoscopic experience do not demonstrate the same ability to carry out a secondary task, suggesting that their technical skills are not fully automated [9].

One practical advantage of attaining technical skill automaticity is that the surgeon is better equipped to deal with distractors common in the operating theater, such as talking, bleeps, phone calls, and external visitors [1, 10]. Another is that the surgeon is more able to attend to cognitively challenging non-technical aspects of a procedure, such as decision making and team communication [11], which can be crucial for surgical competence and patient safety [2]. Unfortunately, automaticity is slow to develop and requires extensive training. One recent study, for example, did not find evidence of expert-like automaticity despite 10 ± 5 hourly sessions of basic laparoscopic skills training over 4 months [12]. Training programs that help surgical trainees to “cheat” some of the time-consuming training needed for technical skill automaticity are desirable [13].

In other skill learning domains, empirical work has validated multitask training as a means to foster qualities of expertise associated with skill automaticity and resilience to stressors that typically disrupt motor performance [14–17]. The approach requires the trainee to practice a motor skill while concurrently conducting a challenging cognitive task. Performance of the task leaves little residual attentional capacity to attend to the motor skill [18] and thus promotes dependence on more automated (implicit) processes to support technical performance. However, a black mark against the practical utility of multitask training is that it tends to slow the rate of learning compared to more traditional single-task training approaches [14, 16, 17].

The overarching aim of the current study was to test the viability of multitask training for laparoscopic skill learning. Specifically, the study aimed to test (1) the relative rate and extent of learning, (2) automaticity, and (3) resilience to stressors of a multitask training intervention compared to a standard single-task training intervention. Multitask training was expected to result in a slower than normal rate of learning, but ultimately multitask trainees were expected to display more signs of automaticity and to be better equipped to deal with common stressors than their conventionally trained counterparts.

## Materials and methods

### Participants

A cluster sample of final year undergraduate medical students (*n* = 106) preparing for objective structured clinical examination (OSCE) volunteered to participate in the study. Participants reported no prior laparoscopy experience. Ethical approval was obtained from the Institutional Review Board, and all participants provided written informed consent. Twenty-five participants withdrew from the study due to scheduling conflicts. Participants were assigned according to their Senior Clerkship rotation group to either a single-task training condition (*n* = 42; 22 males, 20 females; M age = 23.17 ± 1.77) or a multitask training condition (*n* = 39; 28 males, 11 females; M age = 23.03 ± 0.99).

### Task

All participants completed the fundamentals of laparoscopic surgery (FLS) peg transfer task training module developed by the Society of American Gastrointestinal and Endoscopic Surgeons (SAGES) [19].

### Procedure

After viewing an introductory video of the peg transfer task, all participants performed repetitions of the task until they reached a criterion level of proficiency, defined by FLS developers as task completion in 54 s or less on two consecutive trials followed by 10 additional non-consecutive trials at the criterion level [20]. Concurrently, participants in the multitask training condition were required to perform a cognitively demanding tone-counting task for the duration of each peg transfer practice trial. A customized computer program sounded a random sequence of high- and low-frequency auditory tones at a rate of 1 tone per 2000 ms [17]. Participants reported at the end of each practice trial the number of high- and low-frequency tones that they had counted.

On a separate day, participants were reacquainted with the task until two consecutive trials were performed within 54 s [21]. They then completed a series of four three-trial counterbalanced test blocks consisting of a retention test, a secondary task test, a distraction test, and a time pressure test. The secondary task test required concurrent performance of the peg transfer task and a cognitively challenging task, which was a more complex version of the tone-counting task performed by multitask trainees. High- and low-frequency tones sounded at random at an increased rate of one tone per 1000 ms; however, participants were only required to count high-frequency tones. In the distraction test, a telephone situated behind participants began to ring early in the trial and was not attended to by the experimenter until trial completion. Participants were not aware beforehand that the telephone would ring. In the time pressure test, participants’ fastest completion time in training was revealed, and a task completion time target was set that was 20 % quicker that their fastest time. Prior to the retention test block, participants were simply instructed to complete the peg transfer task to the best of their capability as they had done in training.

Finally, all participants performed the same FLS peg transfer task at an OSCE laparoscopic station 1–3 weeks later. Participants were asked to complete as many trials as possible within the 6-min station time limit. All participants completed at least two trials.

### Dependent measures and analysis

The extent of technical skill learning achieved by participants was in the first instance quantified by the number of trials required to meet the proficiency criteria. Three dependent variables were used to evaluate the retention and transfer of laparoscopic performance: task completion time (s); number of hand movements; and hand path length (mm). Throughout the testing session, motion tracking sensors were attached to the dorsum of each hand, and positional data were converted into hand movement and hand path length variables via proprietary software (Imperial College Surgical Assessment Device or ICSAD) [22]. Completion time was measured manually using a stopwatch. To provide an index of the impact of the three transfer conditions on task completion time and movement efficiency (i.e., number of hand movements and hand path length), percentage change from performance in the retention test was calculated for each variable. The time constraints imposed by OSCE did not allow for the setup of the motion tracking system, so completion time was the only performance measure collected. Tone-counting accuracy was calculated as percentage concordance between the number of high tones reported and the actual number presented. The normality of the distribution of data collected for each dependent measure was assessed using Kolmogorov–Smirnov and Shapio–Wilk tests. Based on this analysis, the training groups were compared using an independent samples *t* test if the data had a normal distribution and a Mann–Whitney test if it did not. Significance levels were set at *p* < .05 for all tests.

## Results

### Extent of learning

The number of trials required by participants in the multitask training condition to meet the proficiency criteria was not different from participants in the single-task training condition (Table 1, *U* = 781.50*, z* = −0.36*, p* = .72, *r* = −.04).

**Table 1:** Laparoscopic peg transfer task performance of participants in the single-task and multitask training conditions in training, in the retention test, and in the OSCE

	Single-task training	Multitask training
Number of trials to reach proficiency^a^	24.50 (21.75–32.75)	25 (20–32)
*Retention test*		
Completion time (s)^b^	43.34 (SD = 5.69)	44.26 (5.22)
Number of hand movements^b^	28.84 (SD = 5.24)	29.44 (4.52)
Hand path length (mm)^a^	179.89 (159.66–218.08)	197.49 (171.05–216.79)
*OSCE*		
Completion time (s)^b^	51.48 (6.03)	*M* = 53.46 (5.98)

All tests for differences between the two training conditions were nonsignificant (*p* > .05)

^a^ *M* (SD); ^b^ *Mdn* (*IQR*)

In the retention test, participants in the single-task and multitask training conditions did not significantly differ in the time taken to complete the task (*t*(79) = −0.75, *p* = .45, *d* = .16), the number of hand movements made (*t*(79) = −0.55, *p* = .57, *d* = .07), or the hand path length (*U* = 882.00*, z* = 0.60*, p* = .55, *r* = .07) (see Table 1). The similarity in the time taken to complete the task extended to performance in the OSCE 1–3 weeks later (Table 1, *t*(79) = −1.49, *p* = .14, *d* = .33). Taken together, these findings suggest that participants in the two training conditions acquired similar movement characteristics to complete the laparoscopic peg transfer task at an equivalent rate.^1^

### Automaticity

**Fig. 1:**
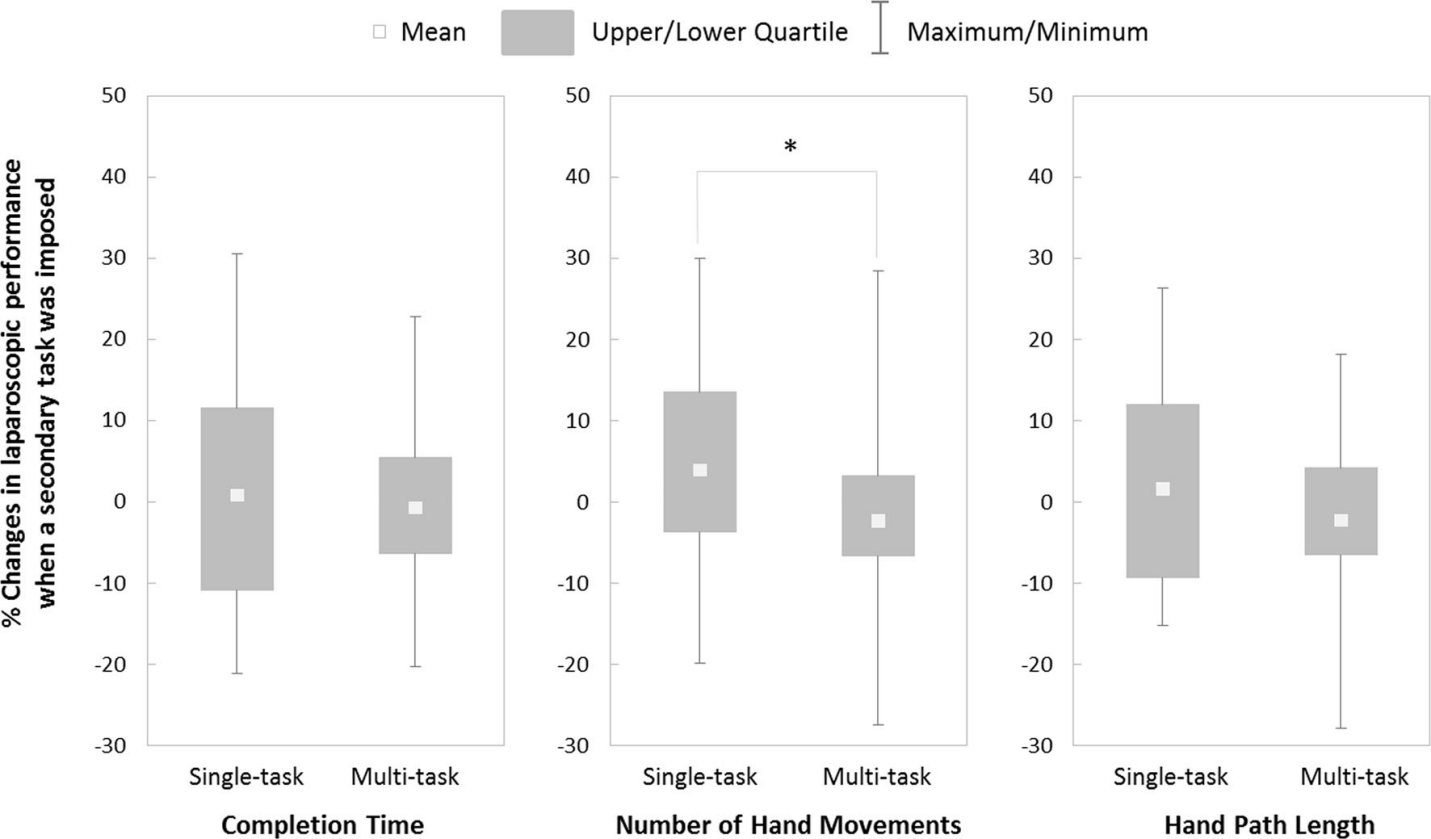
Percentage changes in laparoscopic performance when a secondary task was imposed

The imposition of a concurrent cognitively demanding secondary task^2^ had a significant effect on the number of hand movements used to complete the task (*U* = 563.50*, z* = −2.42*, p* = .016, *r* = −.27), but had no differentiating effect on the hand path length (*U* = 720.00*, z* = −0.94*, p* = .35, *r* = −.10) or the completion time (*t*(79) = 0.58, *p* = .57, *d* = .12). Figure 1 shows that the imposition of a secondary task tended to increase the number of hand movements of participants who had received single-task training, whereas the number of hand movements of participants who had received multitask training was unaffected.

### Resilience

#### Distraction

**Fig. 2:**
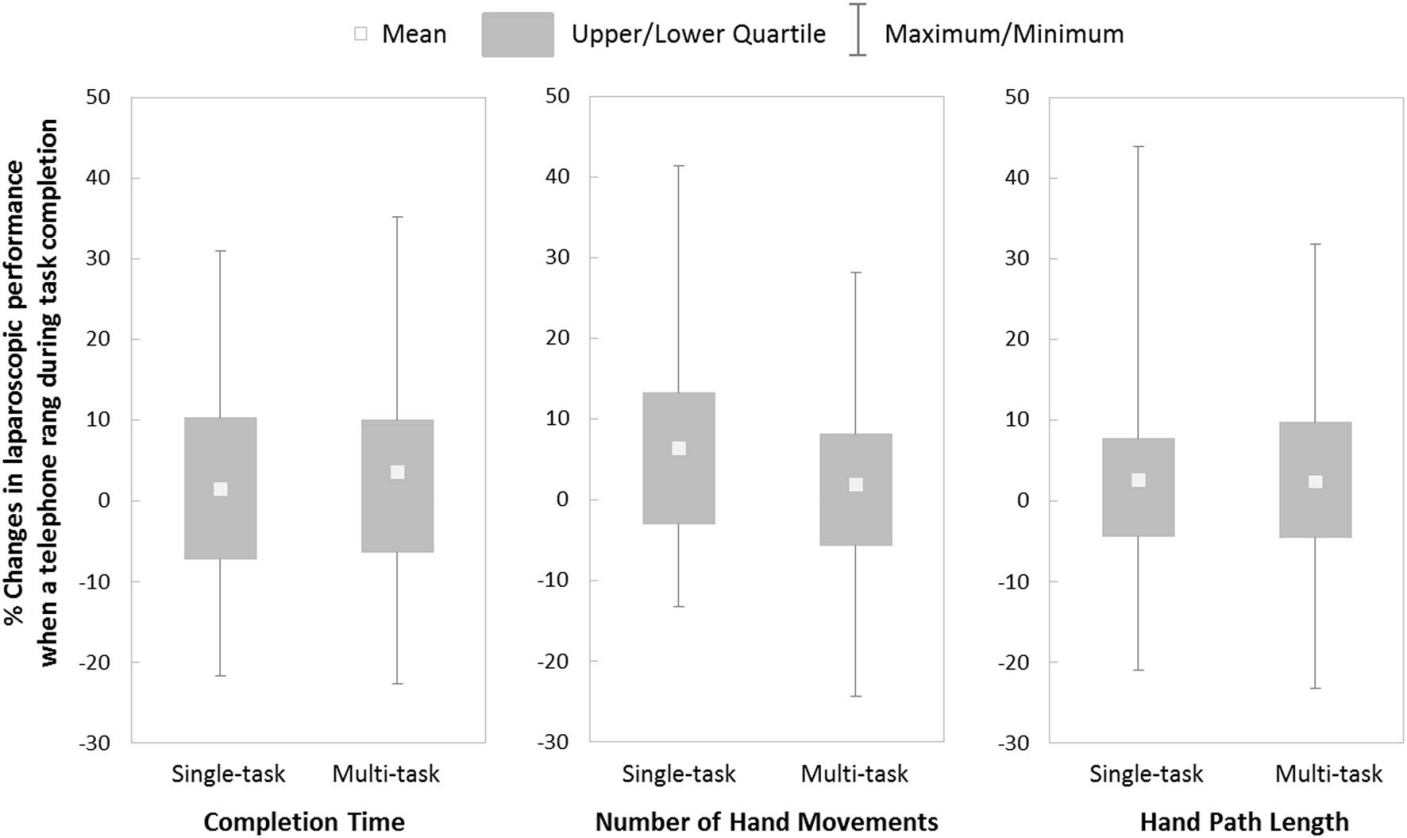
Percentage changes in laparoscopic performance when a telephone rang during task completion

 The unexpected and prolonged sound of a telephone during task completion did not have a differential effect on the completion time (*t*(79) = −0.73, *p* = .47, *d* = .16) or hand path length (*U* = 820.00*, z* < .01*, p* = .99, *r* < .001) of participants in the two training conditions. Observation of the data presented in Fig. 2 suggests that the auditory distraction resulted in more hand movements by participants who had received single-task training than those who had received multitask training. However, the difference was not significant, and the effect size was small to moderate (*t*(79) = 1.58, *p* = .12, *d* = .35).

#### Time pressure

**Fig. 3:**
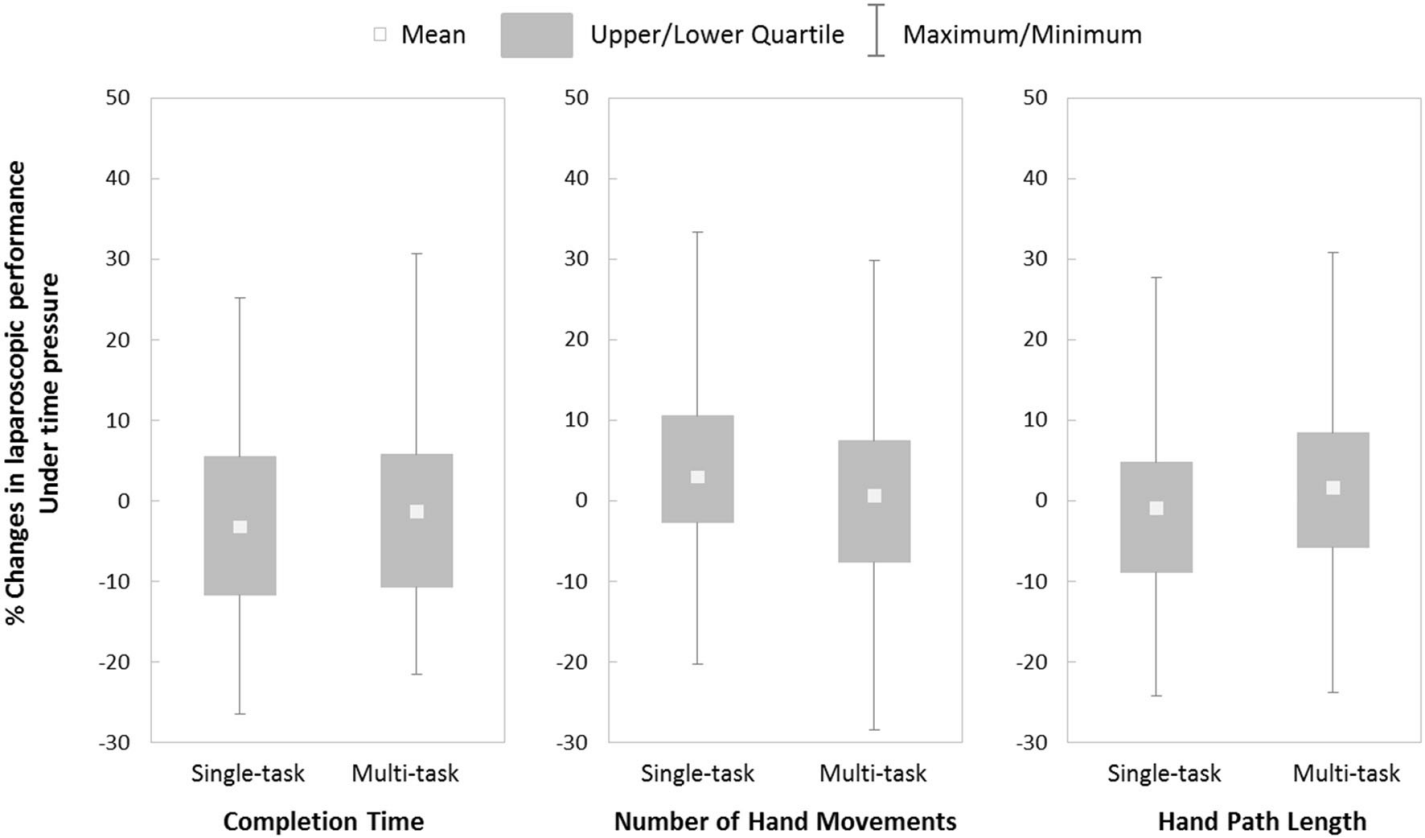
Percentage changes in laparoscopic performance under time pressure

 Application of time pressure did not appear to have a differential effect on the completion time (*t*(79) = −0.70, *p* = .49, *d* = .16), number of hand movements (*t*(79) = 0.92, *p* = .36, *d* = .20), or hand path length (*t*(79) = −0.95, *p* = .34, *d* = .22) in the two training conditions (see Fig. 3).

## Discussion

A study was conducted to examine multitask training of fundamental laparoscopic skills. Recent experimental research outside the surgical domain suggests that asking trainees to practice a primary technical skill while concurrently performing a non-technical secondary task (i.e., multitask training) can be detrimental to the progression of learning, yet can promote beneficial performance characteristics, such as automatic control of movement and resilience to perceived stressors [14–17].

The findings imply that multitasking during training of a fundamental laparoscopic task does not hinder the extent of skill learning. The specified proficiency criterion was reached in the same number of trials as trainees who were free to exclusively attend to peg transfer performance (single-task training condition). Furthermore, performance in training was retained equally in the two training conditions, as demonstrated by equivalent performance in the retention test and the OSCE.

Laparoscopic performance in the two training conditions was affected differently, however, by imposition of a secondary task to evaluate the automaticity of laparoscopic performance. Hand movements of trainees tended to increase in the single-task training condition, but not the multitask condition (Fig. 2), although completion times were not affected differently. The greater number of hand movements by single-task trainees implies that movement efficiency was compromised because they were more dependent on conscious control for effective performance. The unchanged efficiency of the multitask trainees suggests that their training better promoted skill automaticity.

The findings provide evidence of the feasibility and added value of multitask training of fundamental laparoscopic skills. An expert-derived criterion of proficiency was attained after a relatively short period of deliberate practice and was accompanied by the expert-defining attribute of more autonomous movement control. In other words, multitask training appears to make the best use of training time by equipping trainees with fundamental laparoscopic skills that display characteristics of expertise that would normally need more practice to achieve. Given the pressures on surgical educators to adapt their curricula to tackle fiscal constraints, lower resident working hours and reduced teaching time, while ensuring that standards of competence and safety are met [24, 25], multitask training represents a viable training tool.

Theoretically, multitask training could be even more economical for fundamental laparoscopic training. In the present study, multitask trainees were required to complete an irrelevant secondary task throughout laparoscopic training, which left little residual capacity to attend to the motor skill. Further research should examine the feasibility of learning important non-technical aspects of surgical skills (e.g., safety checklists) alongside technical skill aspects, which might otherwise be learnt in the classroom or as part of independent study. Multitask training may also better prepare trainees to deal with the additional cognitive demands of learning more advanced laparoscopic skills.

One widely advocated approach to stress management is to expose trainees to stressors in the safe haven of a simulation-based training environment [2]. This approach is thought to facilitate desensitization by allowing trainees to discover adaptive coping strategies [26] necessary for successful introduction to the operating theater. Preliminary empirical investigation in surgery has confirmed that introducing common stressors (direct observation by an authority figure) into simulation-based laparoscopic training is feasible [26], but the gradual introduction of stressors (elevated procedure complexity, noise distractions) into a training curriculum (FLS model) did not suggest that operative performance (porcine Nissen fundoplication model) was advanced or hindered by stress exposure [9]. For junior surgeons, multitask stressors pose serious threats to surgical performance. Multitask training therefore exposes trainees to a commonly encountered stressor throughout training and may better equip trainees to cope in operating environments that necessitate high-level cognitive involvement in non-technical aspects of a procedure, such as decision making [26].

Surgeons also commonly encounter auditory distractions in theater (e.g., beeps, talking, phones). While not requiring action by the surgeon, such distractions may nevertheless be stressors [1] that impact upon performance. A recent study demonstrated that intraoperative distractors (e.g., external visitors) were associated with reduced completion of safety checklists [10], suggesting that distractions can also compromise non-technical skills. In this study, an unanswered telephone call during laparoscopic performance did not have a significant differential impact on the two groups of trainees, although there was suggestion that single-task trainees needed more hand movements to complete the task.

Laparoscopic performance in the two training conditions was not affected differently by our time pressure manipulation. Any effort to quicken completion time did not appear to result in significantly faster task completions or meaningful changes in movement efficiency. One explanation for the lack of effects could be that setting a target time that was 20 % *faster* than the *fastest* time in training was perceived as unattainable by most trainees. As a result, they may have reduced efforts to achieve the specified goal [27] and reengaged in performing the task to the best of their capabilities [28], as was the requirement throughout training and in the retention test. Inclusion of a self-report workload measure (e.g., SURG-TLX) would provide insight into whether trainees experienced greater temporal demands and/or reduced effort [29, 30].

### Future challenges and limitations

The failure of distraction or time pressure to disrupt the performance by single-task trainees was unexpected and calls into question the validity of our manipulations and limits conclusions about the extent of the resilience of multitask trained skills. Outside the surgical domain, interventions that encourage the use of more automatic (implicit) processes from the onset of learning produce skills that appear resilient to a host of stressors (e.g., ego-threatening feedback, evaluation apprehension, fatigue) (see [31] or [32] for a review) that typically disrupt skills acquired by more conventional (explicit) means (e.g., technical instruction, discovery learning). It is imperative to test the resilience of multitask trained surgical skills in more immersive simulation environments [33] and by exposure to a spectrum of stressors that impact cognitive function, such as fatigue or sleep deprivation [34], heat stress [35], or performance anxiety [36].

Alternatively, it is possible that the dependent measures used in this study failed to fully capture the effect that our manipulations had on laparoscopic performance. Although the ICSAD is an established measure of surgical movement kinematics [25], it may fail to pick up significant, or subtle, movement errors caused by the stressors. Furthermore, no measure of the quality of task completion was recorded. During data collection, we observed participants taking little heed of the forces applied inside the laparoscopic box, which is not advisable when dealing with human tissue. Ratings of task completion quality by experienced surgical educators should be considered in future work.

In this study, medical students’ fundamental laparoscopic skills were trained on a basic laparoscopic task. It remains unclear whether multitask training facilitates the learning of more complex laparoscopic skills (e.g., intracorporeal knot tie) or procedures with a number of crucial non-technical elements. We certainly cannot advocate the application of a multitask intervention for the training of junior surgeons within a live training environment, where attentional resources need to be readily available to deal with the non-technical skill-related challenges of the operation.

It is also unclear whether learning in this study was specific to the peg transfer task (e.g., learning the most time and movement efficient sequence of peg transfers) or the more general acquisition of the visuospatial and movement constraints of laparoscopic tasks. Further empirical investigation is needed to ascertain whether the skill gains achieved in training fundamental laparoscopic skill tasks transfer to the performance of more complex laparoscopic procedures (e.g., use of cross-hand technique) and beyond into the operating theater (see [9]).

Lastly, the effect of multitask training in this study was tested on medical students who possessed no prior laparoscopic experience, so the training benefits may be specific to this level of expertise. The clinical utility of exposing experienced surgeons to bouts of multitask training warrants further investigation.

## Conclusion

We previously contended that surgical educators should consider methods that promote dependence on more automated (implicit) processes as a means of training surgical skills [37–39]. Our findings suggest that multitask training is not detrimental to the rate and extent of surgical skill learning and may promote automatic control of laparoscopic skills. However, it remains unclear whether promoting automaticity also promotes resilience to common stressors experienced by trainee surgeons.

## Footnotes

1 Consistent with recent research [23], the number of trials required by participants in the single-task training condition to reach proficiency was a significant predictor (*F*(1, 39) = 15.73, *p* < .001, with an *R*^*2*^ of .287) of task completion time in the OSCE, implying that laparoscopic ability had a significant bearing on the retention of laparoscopic performance under clinical examination conditions. Interestingly, this was not the case following multitask training, suggesting that a secondary benefit of the training intervention is that it suppresses individual differences. The implication for surgical education is that an individual’s motor competency does not have to be a prerequisite for (self-) selection onto a surgical practice pathway if multitask training is put into practice.

2 The tone-counting accuracy of participants in the single-task training condition (*Mdn* = 97.50 %) and the multitask training condition (*Mdn* *=* 98.00 %) was not significantly different (*U* = 827.50*, z* = 0.08*, p* = 0.94, *r* < 0.01).
